# Surgical management of giant Xp11.2 translocation renal cell carcinoma with multivisceral invasion: a case report of en bloc resection and targeted-immunotherapy success

**DOI:** 10.3389/fonc.2025.1709543

**Published:** 2026-01-14

**Authors:** Bohan Luo, Han Luo

**Affiliations:** 1 School of Medicine, University of Electronic Science and Technology of China, Chengdu, China; 2 Department of Hepatobiliary Surgery, Zigong Fourth People’s Hospital, Zigong, China

**Keywords:** giant renal tumor, multivisceral resection, sunitinib-sintilimab combination, TFE3 gene fusion, Xp11.2 translocation renal cell carcinoma, young adult malignancy

## Abstract

A 36-year-old man presented with a 1-month history of abdominal distension and a 3-day palpable mass. Imaging demonstrated a 20-cm left renal tumor with adjacent organ infiltration and suspicious lymphadenopathy, radiologically suggestive of sarcoma or advanced malignancy. He underwent radical en bloc resection including left nephrectomy, distal pancreatectomy, splenectomy, and partial colectomy due to tumor invasion of the pancreatic tail, splenic vessels, and descending colon mesentery. Histopathology confirmed Xp11.2 translocation/TFE3 gene fusion renal cell carcinoma. Surveillance MRI at 3 months postoperatively revealed local recurrence, prompting combination therapy with sunitinib (VEGF inhibitor) and sintilimab (anti-PD-1 antibody). Follow-up imaging demonstrated significant regression at 1 month, with no evidence of disease progression at 12-month reassessment.

## Introduction

Renal cell carcinoma (RCC) represents approximately 2-3% of all adult malignancies, with clear cell RCC being the most common subtype accounting for 70-80% of cases (1). However, translocation-associated RCCs involving Xp11.2/TFE3 gene fusions are exceedingly rare, comprising only 1-5% of all RCCs and typically presenting in children and young adults (2, 3). These tumors often demonstrate aggressive behavior with advanced disease at diagnosis, including local invasion or metastasis, posing significant diagnostic and therapeutic challenges (4). Here we report a rare case of Xp11.2 translocation/TFE3 gene fusion-associated RCC in a young adult presenting with massive abdominal involvement requiring multivisceral resection.

## Case presentation

A 36-year-old man presented with a 1-month history of abdominal distension and a 3-day history of a self-palpated abdominal mass. He had no significant medical or surgical history, denied tobacco or alcohol use, and reported no family history of hereditary disorders.

## Investigations

**Figure 1:**
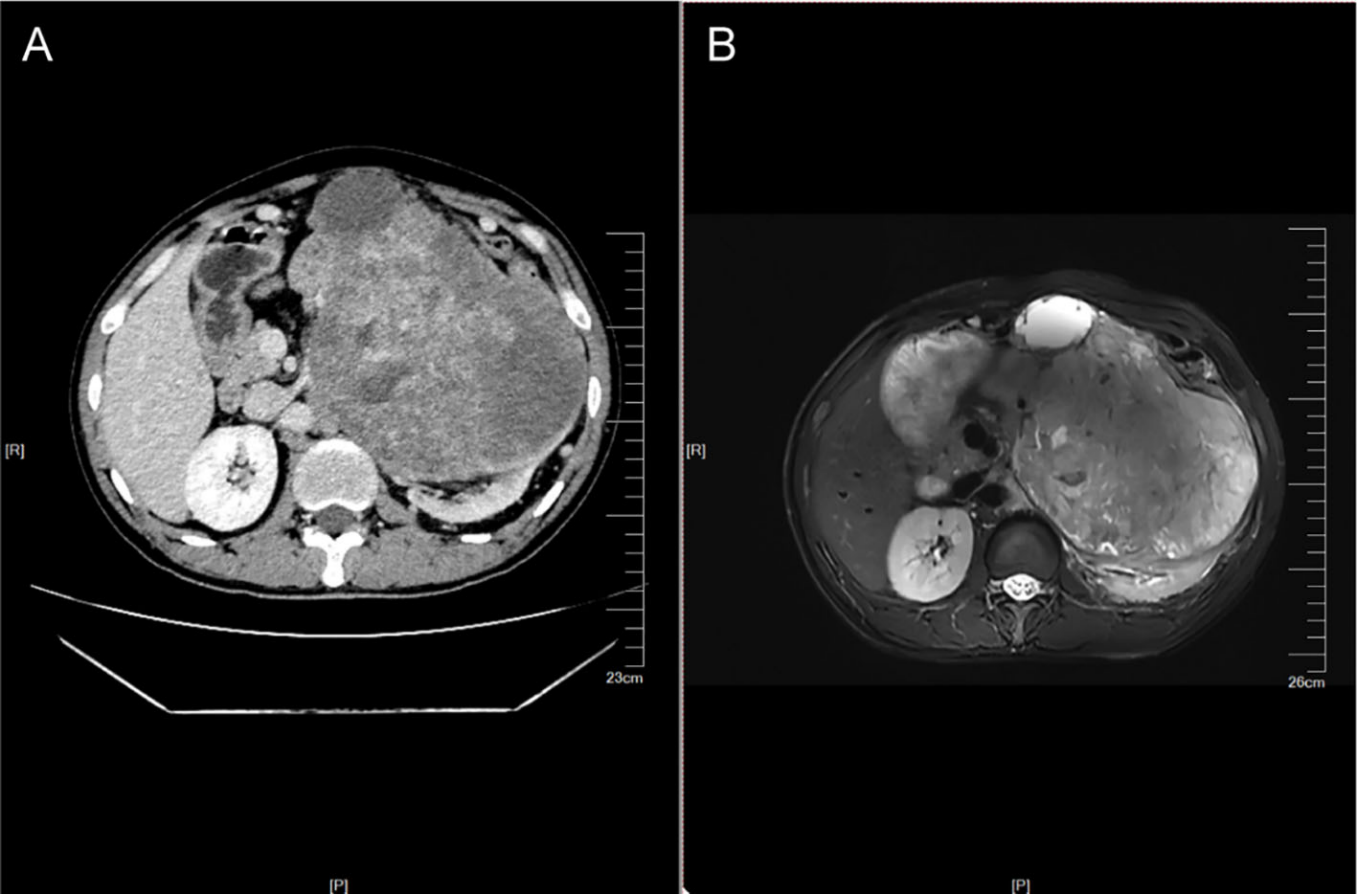
Preoperative imaging examination. **(A)** Contrast-enhanced CT images show the morphology of the mass. **(B)** MRI images show the morphology of the mass.

Physical examination revealed a firm, immobile mass in the left upper quadrant measuring approximately 8 × 6 cm without overlying skin changes. Laboratory studies on admission revealed unremarkable complete blood count and coagulation profiles. Contrast-enhanced abdominopelvic computed tomography (CT) demonstrated an irregular, heterogeneously enhancing mass from the left kidney, suggestive of a primary renal malignancy (**Figure 1A**). The mass exhibited aggressive features, including direct invasion into the pancreatic tail, splenic hilum, and descending colon mesentery. The tumor appeared adherent to the left psoas muscle. No definite tumor thrombus was identified within the left renal vein or inferior vena cava. Multiple enlarged para-aortic lymph nodes were noted, raising concern for metastatic involvement. Magnetic resonance imaging (MRI) with intravenous contrast corroborated these findings, revealing a T2-hyperintense, diffusion-restricted left renal mass with necrotic components and heterogeneous enhancement, further supporting the diagnosis of a high-grade renal neoplasm (**Figure 1B**). No distant metastases were identified in the thorax or skeletal system on baseline imaging. Given the imaging characteristics—including local infiltration and nodal involvement—the multidisciplinary team established a provisional diagnosis of locally advanced left renal malignancy, warranting surgical exploration and resection.

## Differential diagnosis

The differential diagnoses for a 36-year-old male presenting with a left upper quadrant mass and abdominal distension included the following:

### Primary renal malignancies

- Conventional renal cell carcinoma (clear cell/papillary subtypes)
- Sarcomatoid renal carcinoma (suggested by CT/MRI heterogeneity and rapid growth)

### Renal sarcomas

- Leiomyosarcoma or liposarcoma (given tumor size and local invasion)

### Metastatic tumors

- Secondary invasion from gastrointestinal primaries (e.g., colorectal adenocarcinoma)

### Benign renal lesions

- Renal angiomyolipoma (though typically fat-density-positive on CT)
- Complex renal cyst (ruled out by solid enhancement pattern)

### Inflammatory conditions

- Xanthogranulomatous pyelonephritis (mimics malignancy but lacks nodal involvement)

## Treatment

**Figure 2:**
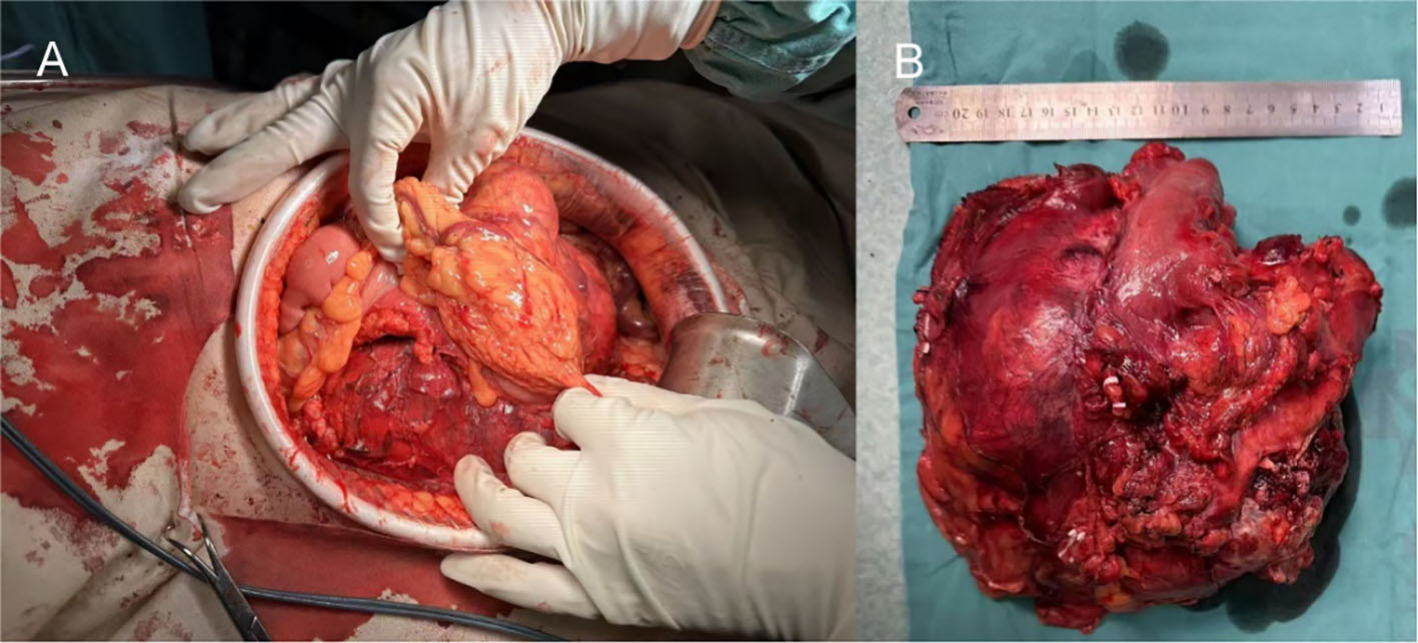
Intraoperative perspective. **(A)** Intraoperative anatomy level. **(B)** Completely resected renal tumor.

Following preoperative imaging, which indicated a left renal mass suspicious for a malignancy with potential metastatic involvement to periaortic lymph nodes, the patient underwent a radical nephrectomy of the left kidney under general anesthesia. Due to extensive adhesions involving the pancreatic body and tail, partial colon, and splenic vessels, the procedure necessitated en bloc resection, including pancreatic body and tail resection with division using a linear stapler and suture reinforcement, splenectomy through ligation of the splenic artery and vein, and partial colectomy. The colon was resected 5 cm proximal and distal to the involved segment, followed by side-to-side anastomosis with staple-line reinforcement (**Figures 2A, B**). Intraoperative hemostasis was achieved with bipolar electrocautery and fibrin sealant application, resulting in a hemorrhage volume of approximately 2000 ml. The significant blood loss was attributed to two main factors: (1) difficulty in hilar dissection due to tumor encasement of the renal vessels, requiring meticulous isolation; and (2) extensive capillary oozing from the broad, inflamed surgical planes created by dissecting the tumor off the adherent pancreas, spleen, and colon. This required transfusion of 5.5 units of packed red blood cells and 300 ml of fresh frozen plasma. Two surgical drains were placed in the retroperitoneal space, and the patient was transferred to the intensive care unit for supportive management, later recovering uneventfully in the ward.

**Figure 3:**
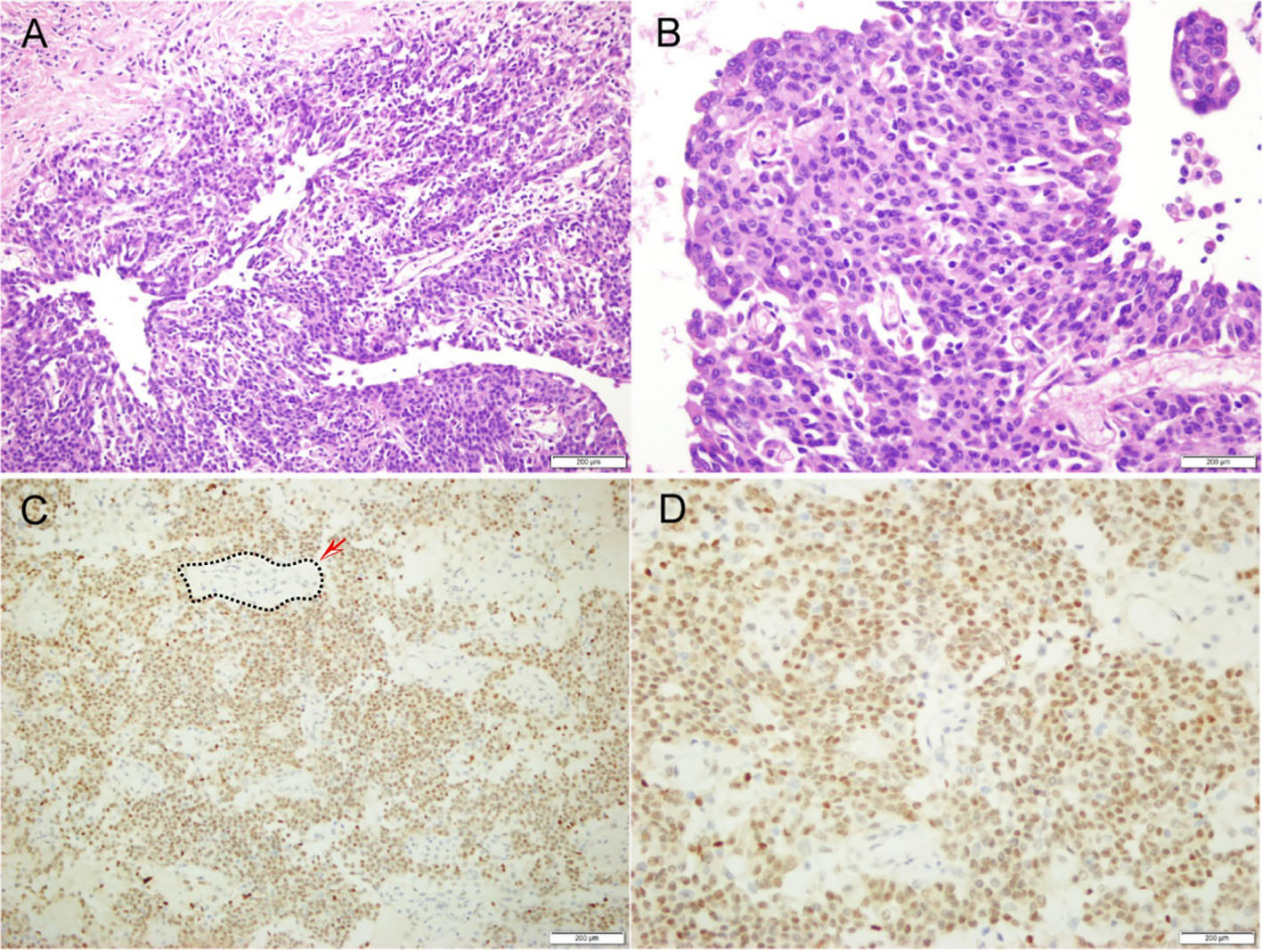
Pathological images. **(A)** 20x HE staining. **(B)** 40x HE staining. **(C)** 20x immunohistochemistry (TFE3). **(D)** 40x immunohistochemistry (TFE3).

Postoperative pathological evaluation confirmed the diagnosis of Xp11.2 translocation/TFE3 gene fusion-associated renal cell carcinoma(**Figure 3**). The tumor was staged as pT3aN0M0 according to the AJCC 8th edition. Microscopic examination confirmed true parenchymal invasion into the pancreatic tail and colonic mesentery, rather than mere adhesion. All surgical margins, including the renal pedicle, pancreatic transection line, and colonic margins, were negative (R0 resection). A total of 15 regional lymph nodes were examined, all of which were negative for metastasis. No tumor capsule rupture was identified intraoperatively.

**Figure 4:**
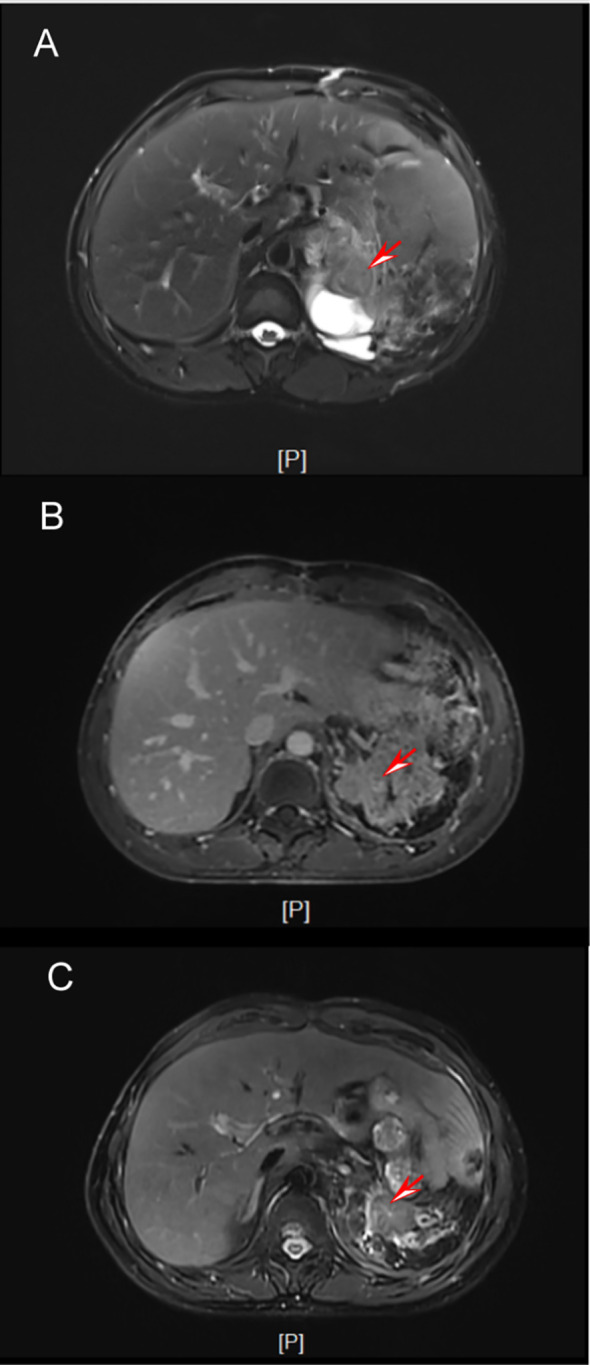
Postoperative follow-up. **(A)** Three months after surgery. **(B)** One month after chemotherapy. **(C)** One year after surgery.

At the 3-month follow-up, MRI surveillance demonstrated a cystic-solid lesion in the surgical field suggestive of recurrence(**Figure 4A**). Based on the immunohistochemical findings and renal cell carcinoma treatment guidelines, a regimen of sunitinib (a multitargeted tyrosine kinase inhibitor) plus an anti-PD-1 antibody was initiated.

The tumor cells outside the black dashed line exhibit diffuse and strong nuclear positivity (brown), while the normal renal tubular epithelial cells inside the black dashed line show negative nuclear staining (blue).

## Outcome and follow-up

At the one-month postoperative follow-up, ultrasonography of the urinary system revealed no abnormalities. Subsequent MRI evaluations at three months postoperatively demonstrated a cystic-solid mass with heterogeneous enhancement in the surgical bed, suggestive of possible neoplastic recurrence, prompting initiation of targeted therapy with sunitinib and sintilimab. Follow-up MRI one month after commencing therapy showed significant reduction in the mass size (**Figure 4B**). By the one-year mark, MRI indicated only postoperative cystic-solid changes without evidence of tumor recurrence (**Figure 4C**). The patient remained clinically stable with no signs of disease progression.

During the treatment period, the patient experienced grade 2 hand-foot skin reaction and grade 1 hypothyroidism, both of which are known common adverse effects of sunitinib. Through symptomatic management (urea-based ointment, temporary drug suspension for one week followed by dose reduction to 37.5 mg/day) and oral levothyroxine sodium tablets, the adverse reactions were well controlled and did not affect the administration of sintilimab. The patient demonstrated good overall compliance with the treatment regimen. No serious adverse events such as immune-related pneumonia, colitis, or myocarditis occurred during the treatment course.

## Discussion

Renal cell carcinoma (RCC) predominantly affects patients aged 60–70 years with tumors <7 cm. This case, however, demonstrates that rare molecular subtypes such as Xp11.2 translocation carcinoma can manifest as massive lesions (>15 cm) in young adults without conventional risk factors (5, 6). The 20 cm left renal tumor in this 36-year-old male exemplifies diagnostic challenges: due to its infiltrative growth and regional lymphadenopathy, initial CT/MRI findings suggested sarcoma. This radiological ambiguity underscores the limitations in preoperatively differentiating translocation RCC from sarcomatoid variants (7, 8).

The surgical complexity—requiring en bloc nephrectomy with distal pancreatectomy and hemicolectomy—reflects the aggressive local behavior of TFE3 fusion carcinomas (9). Encasement of the splenic vasculature by the tumor necessitated triple Hem-o-lok ligation of the renal vessels, while pancreatic transection with a 1 cm margin balanced oncological radicality against the risk of fistula. Despite an estimated blood loss of 2000 mL, this multivisceral approach achieved R0 resection, consistent with outcomes reported for T4 renal malignancies (10).

Pathologically confirmed Xp11.2 translocation RCC, representing <1.5% of adult renal cancers (11), carries distinct therapeutic implications. These molecularly defined tumors exhibit MET pathway activation and intrinsic resistance to VEGF inhibitors (12, 13). The detection of local recurrence by surveillance MRI at 3 months postoperatively aligns with this aggressive biology. Subsequent regression following treatment with sunitinib plus an anti-PD-1 antibody supports evidence of enhanced immunogenicity in fusion-driven carcinomas (14).

For locally advanced renal cancer, as in this case, radical resection remains the preferred treatment option and can significantly improve survival outcomes. CSCO guidelines clearly recommend radical nephrectomy for T3-T4 renal cancer in patients who can tolerate surgery, emphasizing the use of multidisciplinary team (MDT) collaboration to address complex adhesions and potential metastases. The en bloc resection performed here, despite extensive tumor adhesions to the pancreas, splenic vessels, and colon, achieved R0 margins. This underscores how the MDT model can enhance both surgical safety and oncological efficacy, as highlighted in treatment guidelines.

Xp11.2 translocation/TFE3 gene fusion RCC is a rare and aggressive subtype that predominantly affects young adults, often presenting with advanced disease and exhibiting limited response to traditional cytokine-based therapies (15). The phase II ESPN trial established sunitinib as an important therapeutic option for metastatic non-clear cell RCC (which includes this subtype), demonstrating superior response rates and a trend toward longer progression-free survival compared to everolimus (16). However, the efficacy of targeted monotherapy remains suboptimal, with often limited duration of response.

Recent years have seen preliminary evidence of the potential for immune checkpoint inhibitors in Xp11.2 tRCC. A subgroup analysis of the CheckMate 920 trial in advanced non-clear cell RCC reported an objective response rate of approximately 30.8% for the combination of nivolumab and ipilimumab in a cohort that included tRCC cases (17). This finding suggests a subset of patients may benefit from immunotherapy and provides a rationale for exploring immune-based combinations. The synergistic model of ‘VEGF-TKI combined with PD-1/PD-L1 inhibitor, ‘ proven in pivotal phase III trials (e.g., KEYNOTE-426) in clear cell RCC to significantly improve prognosis (18), is now being extended to Xp11.2 tRCC. Although prospective large-scale data are still lacking, similar combinations (e.g., axitinib with PD-1 inhibitors) have been employed in clinical practice with observed positive efficacy (19).

Against this backdrop, we report a young patient with Xp11.2 tRCC who experienced early postoperative recurrence and received combination therapy with sunitinib plus sintilimab. Employing a ‘sunitinib (a VEGF-TKI) combined with sintilimab (a PD-1 inhibitor)’ regimen, the patient achieved sustained remission for over 12 months. Our case shares a mechanistic foundation (VEGF-TKI + PD-1 inhibitor) with the regimens reported in the aforementioned literature and adds new case-based evidence supporting the application of this combination model in Xp11.2 tRCC. The particularity of this case lies in the extremely rapid recurrence (3 months postoperatively), suggesting that such high-risk patients may require early and intensified systemic treatment intervention.

### Consideration of preoperative biopsy and neoadjuvant therapy

Given the tumor’s massive size and multivisceral involvement, the potential role of preoperative biopsy to guide neoadjuvant therapy was discussed within our multidisciplinary team. Unlike Wilms tumor, where neoadjuvant chemotherapy is standardized, there is currently no established, consistently effective neoadjuvant regimen for adult Xp11.2 tRCC. Furthermore, the risk of tract seeding and hemorrhage from a biopsy of such a large, hypervascular tumor was deemed significant. The primary goal at presentation was prompt cytoreduction for symptomatic relief and definitive diagnosis via surgical pathology. This case highlights an unmet clinical need. As evidence for targeted and immunotherapy in advanced tRCC accumulates, future similar presentations may warrant image-guided biopsy to explore the possibility of neoadjuvant systemic therapy, which could potentially downstage the tumor and facilitate less morbid surgery.

### Treatment and follow-up plan

**Figure 5:**
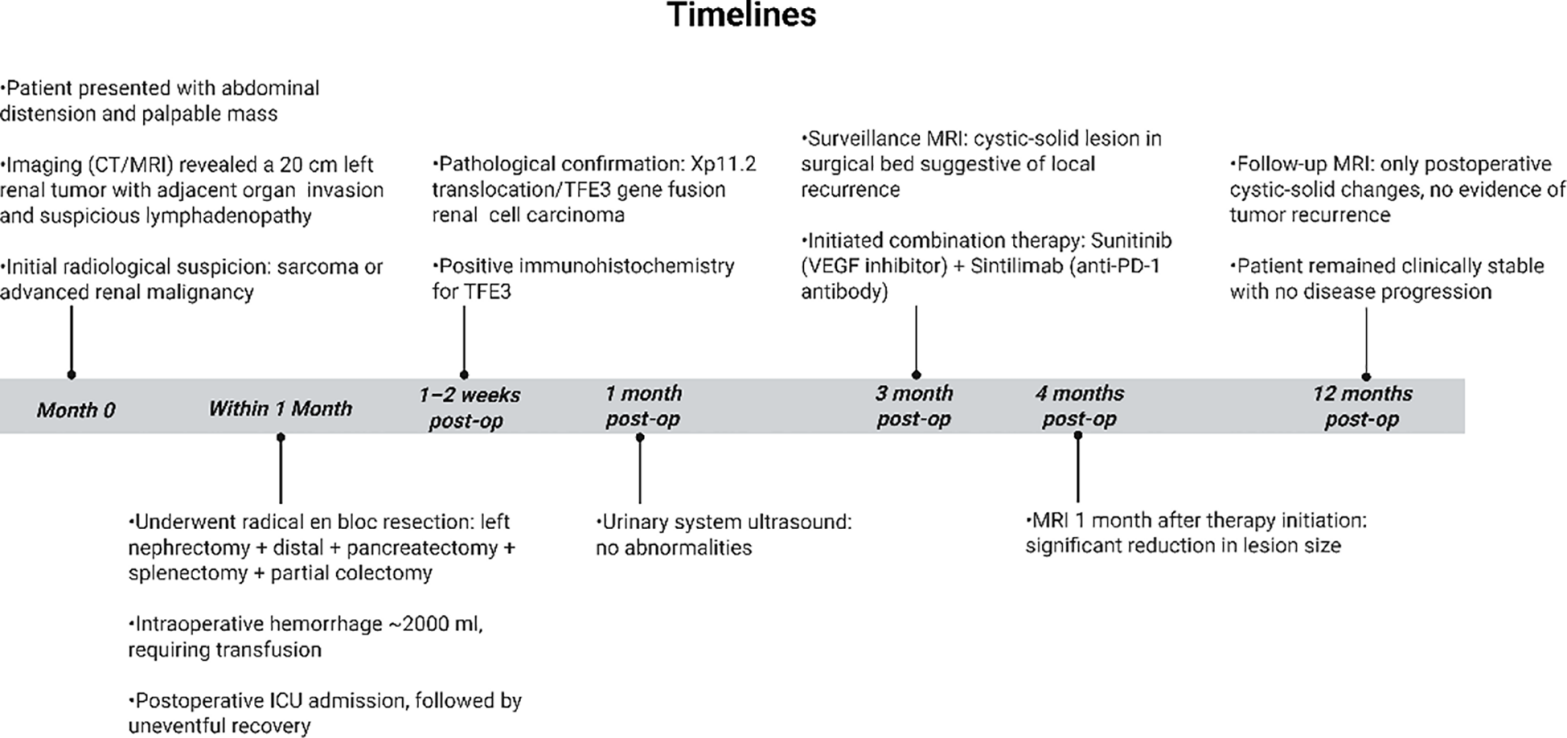
Timelines.

The patient continues treatment with sunitinib and sintilimab. Comprehensive evaluations, including chest and abdominal CT scans, are scheduled every 3 months. If a sustained complete or partial response is maintained for over 2 years, we will consider discussing with the patient the possibility of attempting a temporary pause in immunotherapy while maintaining close monitoring. Regarding potential drug resistance, our plan is that if disease progression occurs, we will first consider switching to a VEGF-TKI with a different mechanism of action (such as benmelstobart plus anlotinib), potentially continuing in combination with immunotherapy (20). If progression persists, a re-biopsy would be considered to explore resistance mechanisms (e.g., genetic alterations, tumor microenvironment changes) and to guide subsequent treatment options, such as clinical trial enrollment (**Figure 5**).

### Limitations

This study has limitations. The diagnosis was primarily based on typical clinicopathological features and positive TFE3 immunohistochemistry. Due to the patient’s personal financial reasons, they declined further self-pay confirmatory testing (such as FISH); therefore, direct molecular evidence of gene rearrangement was not obtained. Nevertheless, the existing evidence strongly supports the diagnosis. Moreover, the core value of this case report lies in documenting a successful clinical experience with combination therapy for this rare and aggressive subtype.

## Patient perspective

The period from discovering the tumor, undergoing major surgery, to experiencing such a rapid recurrence was incredibly difficult for me. However, after starting the combination therapy, my condition stabilized quickly. Now, the results of every follow-up examination are reassuring, my body feels stronger, and I have largely returned to normal daily life. I have great confidence in this treatment plan and will continue to cooperate with my doctors by adhering to the therapy and attending all follow-up appointments.

## Learning points

- Young adults presenting with massive renal tumors (>15 cm) warrant comprehensive surgical planning, as their size often correlates with adjacent organ invasion and complex multi-visceral resection requirements.
- XP11.2 translocation renal cell carcinoma, though exceedingly rare in adults, necessitates aggressive en-bloc resection of involved organs (e.g., pancreas, spleen, colon) due to its infiltrative behavior—highlighting the critical role of multidisciplinary intraoperative decision-making.
- Histopathological diagnosis of TFE3-gene fusion tumors mandates immunohistochemical confirmation, as this entity dictates distinct therapeutic pathways compared to conventional renal carcinomas.
- Sustained tumor control in advanced translocation RCC may be achieved through combined VEGF-targeted therapy (e.g., sunitinib) and immune checkpoint inhibition, even following suboptimal resections with positive margins.

## Data Availability

The original contributions presented in the study are included in the article/supplementary material. Further inquiries can be directed to the corresponding author.
